# Supplementary material for: Wnt ligands influence tumour initiation by controlling the number of intestinal stem cells
Source: Nat Commun. 2018 Mar 19;9:1132. doi: 10.1038/s41467-018-03426-2 (PMC5859272; doi:10.1038/s41467-018-03426-2)
Supplement: Supplementary file 1 — Supplementary Information(PDF 2056 kb) [file 41467_2018_3426_MOESM1_ESM.pdf]

Supplementary Information

Supplementary Figure 1

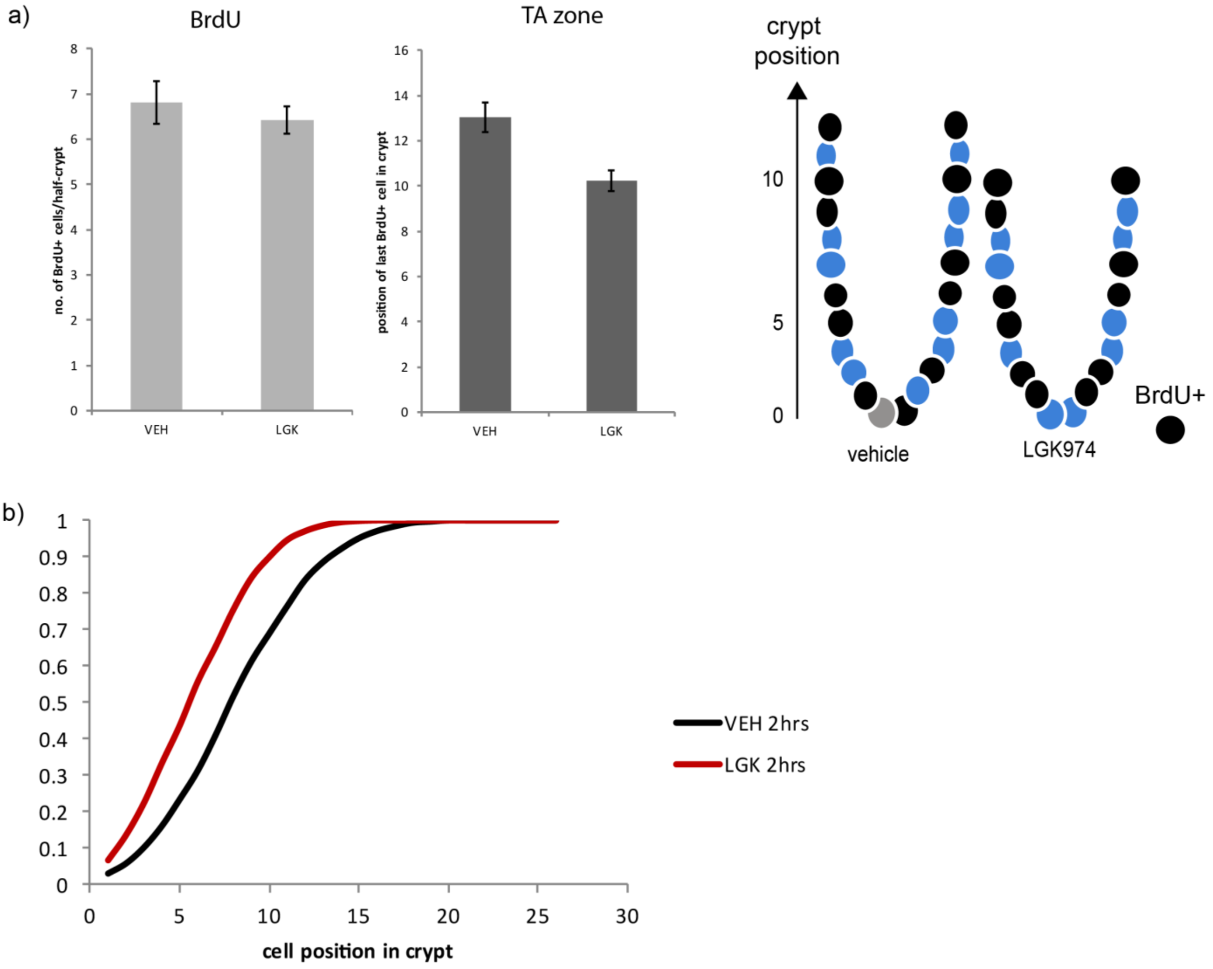

Supplementary Figure 1. Porcupine Inhibition changes size of proliferative zone. a) Although the number of BrdU+ cells per crypt did not change, there is a reduction in the height of the proliferative zone (TA zone). The last BrdU+ cell in vehicle treated mice is about +13, whereas after LGK974 treatment the last positive BrdU+ cell is at position +10 (N=3 mice per group). b) Scoring of BrdU+ cells per position reveals the distribution of the proliferative cells along the crypt-villus axis. For example, whereas 50% of all proliferative cells in the vehicle mice are found until position +8, 50% of all the proliferative cells are found until position +5 (N=3 mice, at least 30 crypts per mouse analysed).

Supplementary Figure 2

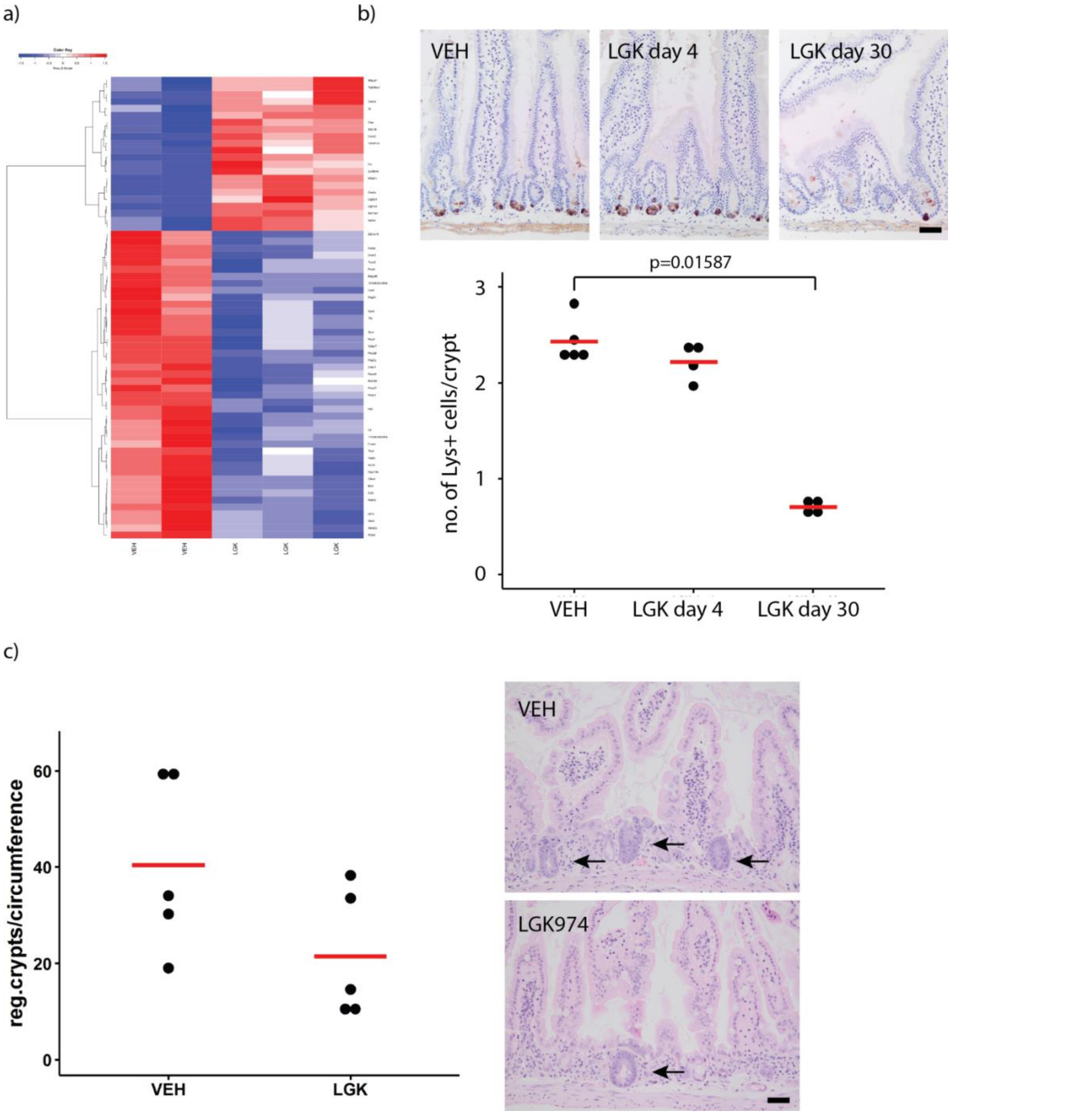

Supplementary Figure 2. Changes of Porcupine treatment on homeostasis and regeneration. a) Heatmap of Pearson correlation of significantly de-regulated genes after LGK974 treatment. Whole small intestinal tissue (proximal small intestine) was analysed. There were 22 genes upregulated (red), and 44 genes downregulated (blue), N=2 vehicle , N=3 LGK974 (see also Supplementary Table 1) b) The number of Paneth cells were scored by lysozyme IHC. Graph depicts mean per mouse of lysozyme+ cells per crypt (100 crypts per mouse scored, N=5 vehicle (treated for 4 days), N=4 LGK974 (treated for 4 and 30 days), statistics: Mann-Whitney U. Scale bar = 50µm. c) Whole body irradiation (10Gy) was performed and treatment with LGK974 started after 6 hrs, mice were sampled 72h post irradiation. Regenerating crypts (arrow) per small intestinal circumference were scored, at least 10 circumferences per mouse were analysed. Each dot represents the mean per mouse; the red line indicates the mean per group. N=5 mice per group, Scale bar = 50µm.

Supplementary Figure 3

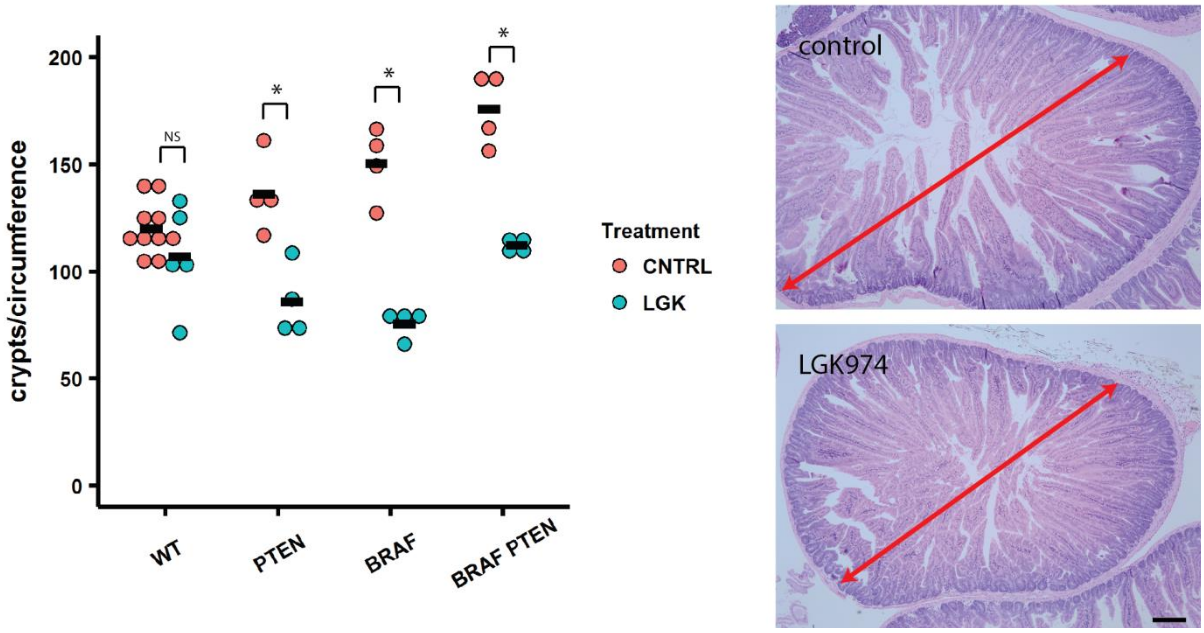

Supplementary Figure 3. Increased crypt number in BRAF and BRAF PTEN mice is dependent on Wnt ligands. The number of crypts per circumference in the small intestine was quantified. At least 10 circumferences per mouse were scored; each dot represents mean per mouse. Wildtype (WT), *VilCre^{ER} Braf $^{V600E/+}$* (BRAF), *VilCreER Pten $^{fl/fl}$* (PTEN) and *VilCre^{ER} Braf $^{V600E/+}$ Pten $^{fl/fl}$* (BRAF PTEN) were sampled 30 days after induction. The H&E show the circumference of a BRAF PTEN mouse untreated (control) or treated with LGK974 (starting 1 day after induction). The red arrow indicates difference in crypt diameter. Scale bar = 100um. Statistics: Mann-Whitney U test between CNTRL and LGK treatment, WT (CNTRL: N=10, LGK: N=5): p=0.371, PTEN, BRAF and BRAF PTEN (N=4 in each group): p=0.02857 (*).

Supplementary Figure 4

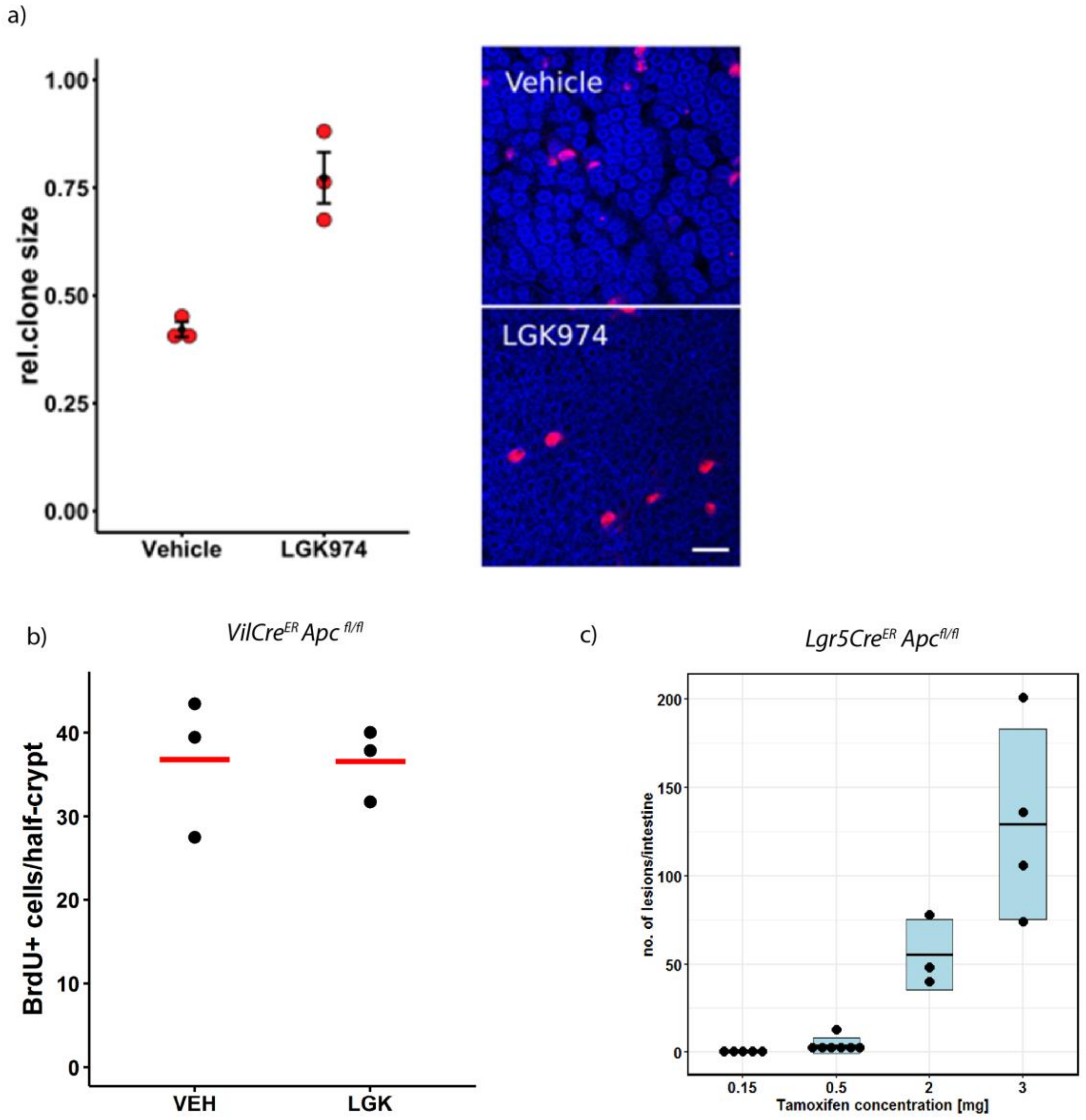

Supplementary Figure 4. Effect of Porcupine treatment on stem cells and proliferation. a) *AhCre*^{ER} *tdTom*$^{fl/+}$ *Apc*$^{fl/+}$ mice were induced with 1mg β-Naphthoflavone and 0.15 mg tamoxifen and treated with Vehicle/LGK974 24hrs after induction until sampling. The base of the crypt was analysed 10 days after induction. For each mouse, >=60 clones from the proximal small intestine were scored for the average clone size. Each red dot represents the mean for each mouse (N=3); the crossbar is mean per group +/- s.e.m. Representative images, tdTom (red), DAPI (blue), scale bar = 100 µm b) The number of BrdU+ cells per half-crypt (at least 30 crypts per mouse) was scored. Each dot represents the mean of one mouse, red bar indicates mean per group. N=3 for each group. Mann-Whitney U test, p=1. c) *Lgr5Cre*^{ER} *Apc*$^{fl/fl}$ (untreated) were induced with different concentrations of tamoxifen (IP) and sampled at 100 days p.i. or when signs of intestinal adenomas were apparent. Data show the number of lesions on a single H&E section of the full length of the small intestine. Each dot represents a single mouse, the box is constructed by the mean +/- standard deviation.

Supplementary Figure 5

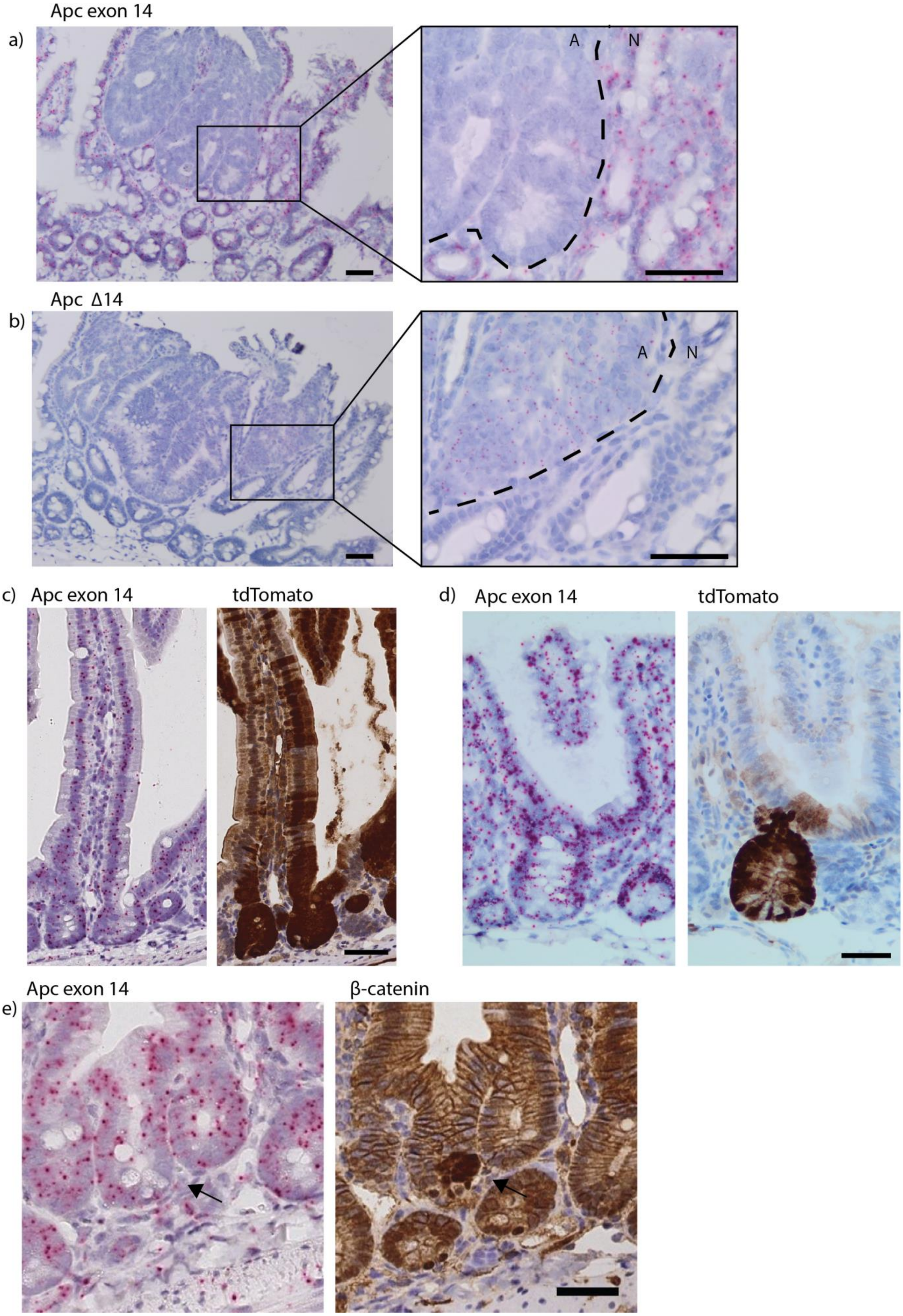

Supplementary Figure 5 RNA *in situ* probe to detect recombined *Apc* allele.

a) RNA *in situ* probe for the wildtype *Apc* exon 14 allele (red dots) and b) RNA *in situ* probe (red dots) for the recombined allele (Δ14) in an established adenoma of a *Lgr5Cre*^{ER} *Apc*$^{fl/fl}$ (3mg tamoxifen). Inserts show specificity of probes between adenoma (A) and normal tissue (N). c) Immunohistochemistry for tdTom (RFP) staining and RNA *in situ* for the deletion of *Apc* on serial sections from *Lgr5Cre*^{ER} *Apc*$^{fl/fl}$ mice induced with 3mg tamoxifen, day 10 p.i. d) Low-level induction in *AhCre*^{ER} *Apc*$^{fl/fl}$ *tdTom* $^{fl/fl}$ mice with 0.15mg tamoxifen and 1mg β-naphthoflavone at day 21. Scale bars = 50µm. e) Loss of Apc exon 14 coincides with accumulation of nuclear β-catenin (arrow - *Lgr5Cre*^{ER} *Apc*$^{fl/fl}$ day 10 p.i, 3mg tamoxifen).

Supplementary Figure 6

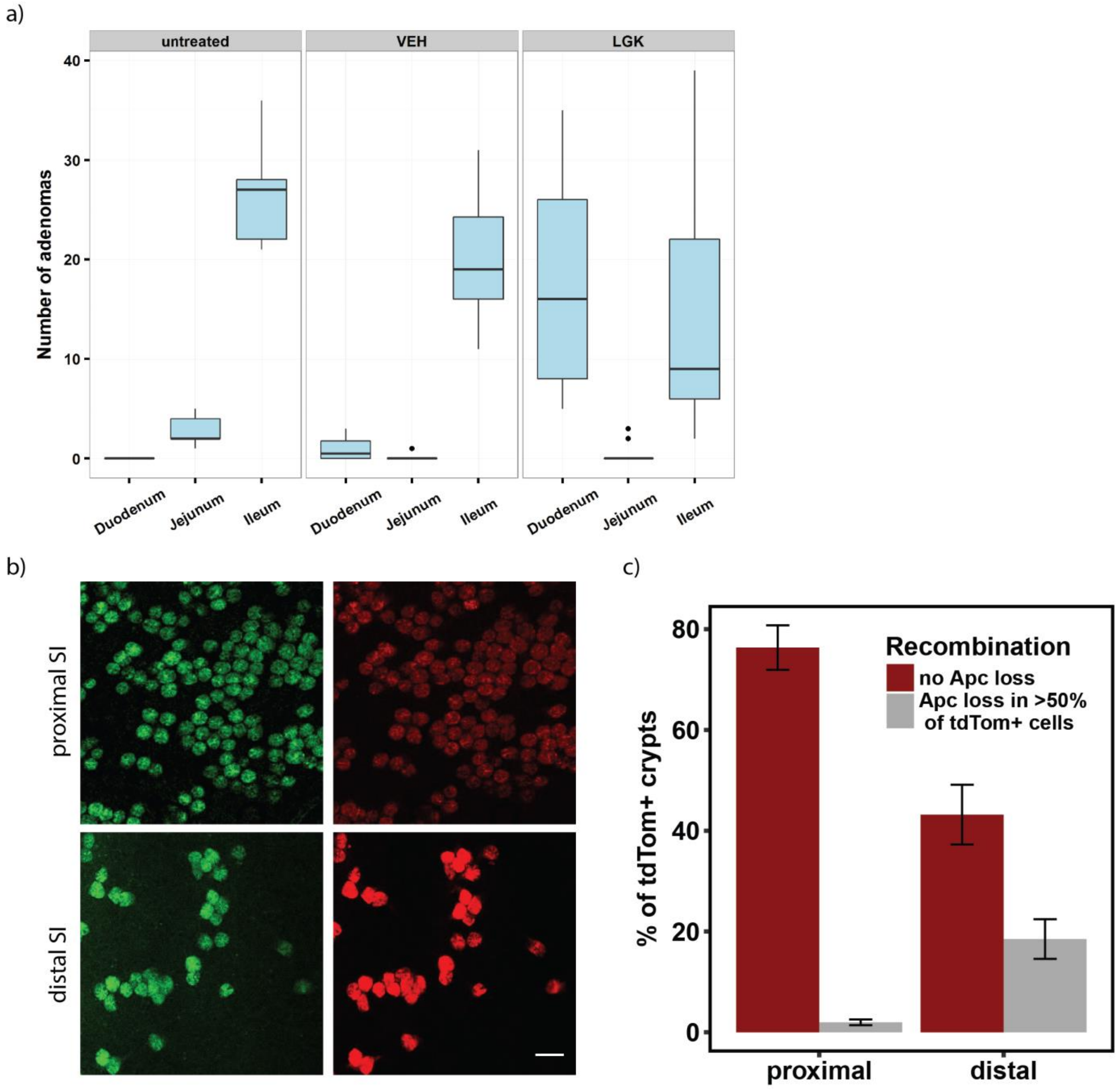

Supplementary Figure 6. **Effect of Porcupine treatment on adenoma distribution and discrepancy between tdTom reporter and *Apc* recombination.** a) *Lgr5Cre^{ER} Apc$^{fl/fl}$* mice were induced (3mg tamoxifen) and sampled when signs of intestinal adenoma burden were apparent. The small intestine was equally divided into 3 sections, from proximal to distal (duodenum, jejunum and ileum) and the number of adenomas per section was quantified microscopically. Note the increased number of adenomas specifically in the duodenum after LGK974 treatment. b) *Lgr5CreER tdTom^{fl}* mice induced with 3mg tamoxifen (IP) were analysed at day 3 p.i. All Lgr5-GFP postitive crypts were fully labelled by tdTom. Scale bar = 100 um. c) *Lgr5Cre^{ER} Apc$^{fl/fl}$* mice, induced with 3mg tamoxifen and sampled day 10 p.i. Serial sections were stained for tdTom (RFP) and RNA *in situ* for *Apc* loss show that the majority of tdTom+ crypts still express Apc. Note, the increased efficiency of Apc deletion in the distal SI (Ileum). N=3 mice with >=100 crypts scored per region per mouse, error bars = s.e.m.

Supplementary Figure 7

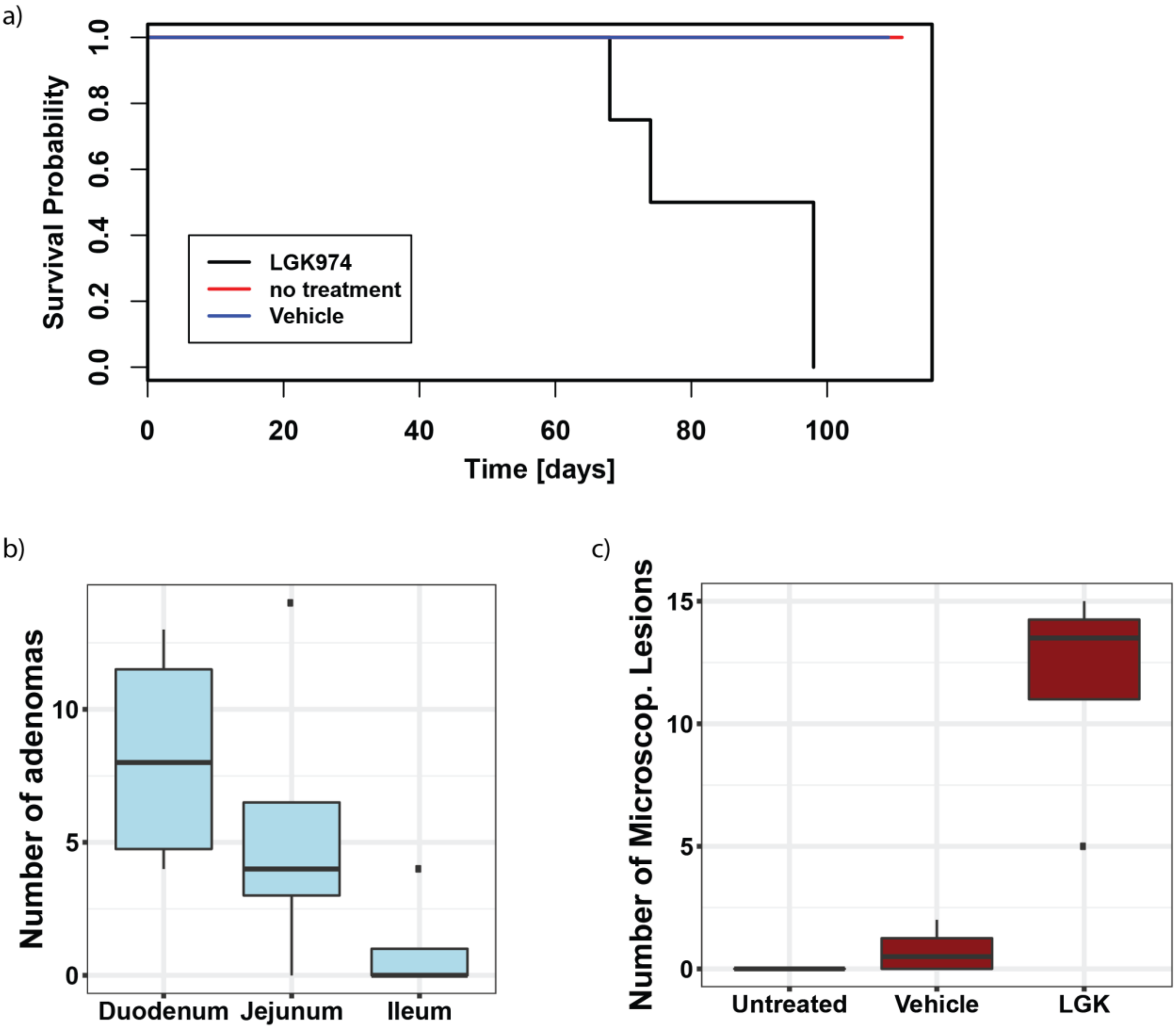

Supplementary Figure 7.Increased tumourigenesis in *VilCre^{ER} Apc$^{fl/+}$* mice after Porcupine treatment. *VilCre^{ER} Apc$^{fl/+}$* mice were induced with 0.15mg tamoxifen (IP) and treated with LGK974 or vehicle from day 1 p.i. continuously. a) Mice were sampled when signs of intestinal adenoma burden were apparent. All remaining mice were finally sampled at day 100-105 p.i. b) The small intestine was arbitrarily divided in 3 equal parts and the number of macroscopically visible adenomas per mouse was counted. Only LGK974 treated mice had visible adenomas. c) Microscopic analysis showed only very few small adenomas in vehicle treated mice, whereas LGK974 had several adenomas per section. N=5 for untreated and vehicle treated mice, N=4 for LGK974 treated mice The boxplots show the median (black line) and the first and third quartiles (box).

Supplementary Figure 8

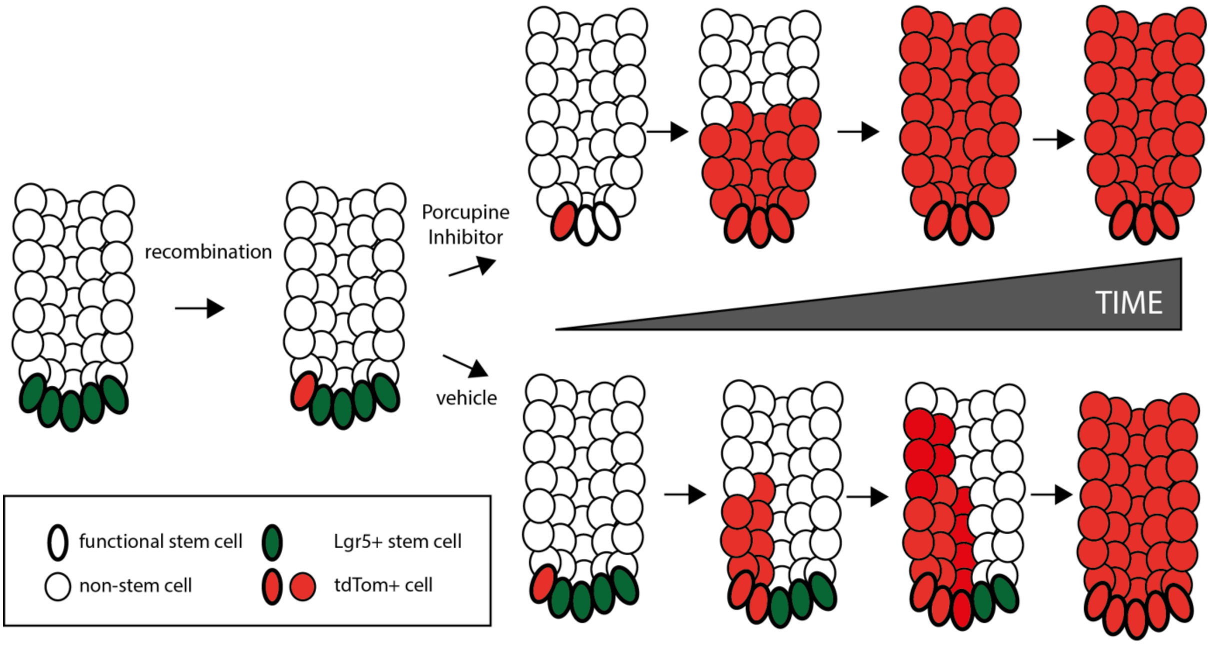

Supplementary Figure 8 Overview of changed stem cell dynamics after Porcupine inhibition. After recombination in a limited number of intestinal stem cells, treatment with Porcupine inhibitor LGK974 reduced the number of functional stem cells in the crypt. The labelled intestinal stem cell competes with the reduced number of intestinal stem cells, which have lost Lgr5 expression, resulting in a quicker fixation of the tdTom+ clone. The same principle applies to loss of Apc in few intestinal stem cells instead of tdTom labelling.
