# Supplementary material for: Wnt ligands influence tumour initiation by controlling the number of intestinal stem cells
Source: Nat Commun. 2018 Mar 19;9:1132. doi: 10.1038/s41467-018-03426-2 (PMC5859272; doi:10.1038/s41467-018-03426-2)
Supplement: Supplementary file 3 — Description of Additional Supplementary Files(PDF 166 kb) [file 41467_2018_3426_MOESM3_ESM.pdf]

Description of Additional Supplementary Files

File Name: Supplementary Data 1
Description: Raw reads of the significantly deregulated genes between vehicle and Porcupine inhibitor treated mice.
